# OASIS: Feasibility Study Protocol of an AI-Based VR Intervention for Adults with Anorexia Nervosa

**DOI:** 10.1192/bjo.2026.11204

**Published:** 2026-06-30

**Authors:** Wafa Alharbi, Hubertus Himmerich, Janet Treasure, Danielle Glennon, Sarah Odoi

**Affiliations:** 1 King’s College London, London, United Kingdom; 2 Taibah University, Madinah, Saudi Arabia; 3 South London and Maudsley NHS Foundation Trust, London, United Kingdom

## Abstract

**Aims:**

Anorexia nervosa (AN) is associated with high morbidity and mortality and significant barriers to recovery, including food-related anxiety and avoidance behaviours. Virtual reality (VR) interventions offer immersive, safe exposure to feared stimuli and may enhance engagement with treatment. The OASIS feasibility study aimed to evaluate the acceptability, feasibility, and preliminary effectiveness of a VR-based, AI-personalized adjunct to treatment as usual (TAU) in adults with AN.

**Methods:**

This is a single-site feasibility study conducted at South London and Maudsley NHS Foundation Trust (SLaM). Up to 45 adults with AN from inpatient, day-care, and outpatient services will participate in a one-week VR intervention alongside TAU. The OASIS app, delivered via Pico 4 headset, will provide personalized sessions including psychoeducation, graded food exposure, motivational support from a virtual companion, and relaxation exercises. Primary outcomes will include recruitment, adherence, retention, and acceptability. Secondary outcomes will include anxiety, relaxation, BMI, and qualitative feedback from focus groups with patients, carers, and clinicians. Data will be analysed descriptively, and thematic analysis will be applied to qualitative data.

**Conclusion:**

The OASIS study will provide evidence on the feasibility and acceptability of a VR-based intervention for adults with AN and will inform the design of a future randomized controlled trial. Insights from patient, carer, and clinician feedback will guide refinement of the intervention and identify barriers and facilitators to implementation in routine clinical care.

